# The impact of steroid-impregnated polypropylene mesh on foreign body reaction in an experimental rat study

**DOI:** 10.1590/acb412926

**Published:** 2026-07-17

**Authors:** Hatice Yilmaz Dogru, Akgul Arici

**Affiliations:** 1 Mersin City Training and Research Hospital – Department of Obstetrics and Gynecology – Mersin – Turkey.; 2 Tokat Gaziosmanpaşa University – Department of Pathology – Tokat – Turkey.

**Keywords:** Steroids, Polypropylenes, Foreign-Body Reaction, Wound Healing

## Abstract

**Purpose:**

Foreign body reaction is a tissue response in which excessive reactions may lead to complications such as chronic inflammation, mesh contraction, and impaired tissue integration. The present study aimed to evaluate the effects of steroid-impregnated polypropylene mesh on foreign body reaction and fibrosis in a rat abdominal wall model.

**Methods:**

Forty adults female Wistar rats were randomly assigned into four groups (n = 10 each): polypropylene mesh in the acute period (group A), steroid-impregnated polypropylene mesh in the acute period (group AS), polypropylene mesh in the chronic period (group C), and steroid-impregnated polypropylene mesh in the chronic period (group CS). Standardized 1 × 1-cm meshes were implanted on the right rectus abdominis muscle. Acute groups were assessed at three days post-implantation, and chronic groups at nine weeks. Histopathological evaluation included grading of polymorphonuclear leukocyte infiltration, neovascularization, foreign-body giant cell reaction, muscle invasion, necrosis, and fibrosis. Immunohistochemical staining for CD15, CD68, CD3, and CD20 was performed to characterize inflammatory cell subtypes.

**Results:**

The mean grading of polymorphonuclear leukocyte infiltration, neovascularization, foreign-body reaction, muscle invasion, and necrosis showed no difference among groups, in which fibrosis levels were significantly higher in group C and group CS compared to group A (*p* = 0.004, *p* = 0.004, respectively). The fibrosis levels were also elevated in group CS than in group AS (*p* = 0.001, *p* = 0.001, respectively).

**Conclusion:**

Local corticosteroid impregnation of polypropylene mesh slightly attenuated early inflammatory responses but did not prevent chronic fibrosis.

## Introduction

Polypropylene mesh is the most widely used synthetic graft material across numerous surgical procedures due to its favorable characteristics, including ease of application, flexibility, promotion of cellular ingrowth, and relatively low cost^1^. Following implantation, polypropylene—like other foreign materials—elicits a marked inflammatory response that facilitates firm integration of the mesh into surrounding tissues. However, this inflammatory reaction may also promote adhesion formation, a significant postoperative concern and an active area of investigation in surgical research^1,2^.

The foreign body reaction represents a complex tissue response triggered by implanted biomaterials and characterized by a cascade of inflammatory events that can, in some cases, become excessive. To minimize this exaggerated response, several investigators have explored modifications to the chemical and physical properties of mesh materials, particularly through surface coatings. Such modifications aim to enhance biocompatibility and optimize mesh–tissue integration^3,4^.

Despite its advantages, polypropylene is recognized for inducing a strong host inflammatory reaction that contributes to adhesion formation and fibrosis around the implant5. Although adhesions constitute a natural component of wound healing, sustained or excessive inflammation may result in complications such as mesh contraction, chronic pain, and impaired tissue incorporation.

To counter these adverse effects, various anti-adhesive strategies—including liquid barriers (*e.g.*, Sepracoat, icodextrin) and coated meshes—have been developed. These interventions are designed either to provide temporary separation of traumatized tissues or to establish a hydrophilic, biologically inert interface that attenuates immune activation^3-5^. Mesh coatings may include corticosteroids, antibiotics, hydrogels, silver, or synthetic polymers, each targeting specific mechanisms underlying inflammation, infection, or fibrosis.

Particularly, corticosteroids possess well-established anti-inflammatory, anti-proliferative, and immunosuppressive properties. They reduce leukocyte migration, inhibit phospholipase A2 activity and downstream eicosanoid synthesis, suppress pro-inflammatory cytokine transcription, and induce vasoconstriction5,6. Given that mesh-induced inflammation is closely associated with foreign body granuloma development and collagen deposition, corticosteroid-based coatings are intended to modulate early inflammatory overactivation and improve long-term biocompatibility. Recent studies have increasingly focused on the influence of corticosteroids on tissue remodeling and fibrosis in the context of foreign body reactions^2,5^.

In this context, the present study employed a rat model to investigate the effects of steroid-coated polypropylene mesh on the progression of inflammation and the severity of fibrosis at both early (three days) and late (63 days) postoperative intervals.

## Methods

### Chemicals, mesh materials, and coating

Ketamine and xylazine were purchased from Alfasan International B.V. Hematoxylin Harris and eosin Y 1% alcoholic were obtained from Atom Scientific Ltd. (Manchester, United Kingdom). Masson’s trichrome was obtained from GBL, Istanbul, Turkey. Antibodies against CD3, CD15, CD20, and CD68 antibodies were obtained from KiMERA Medical Laboratory Supplies, Istanbul, Turkey.

### Animals

After obtaining approval from the Animal Experiments Local Ethics Committee (Protocol No.: 2016-HADYEK-03), a total of 40 nulligravid female Wistar–Albino rats (*Rattus norvegicus*) weighing 250–350 g and aged approximately 10–12 weeks old were randomly selected for the study from the breeding colony of the Tokat Gaziosmanpasa University Experimental Medicine Research Unit. All experiments were conducted in Tokat Gaziosmanpasa University Experimental Medicine Research Unit laboratories. Randomization was performed using a computer-generated sequence to minimize selection bias.

All animals were housed in a controlled laboratory environment maintained at 20–24°C, with a 14-h light / 10-h dark photoperiod (lights on from 6 to 8 p.m.) and a constant 40–60% relative humidity. Environmental conditions were monitored twice daily. The rats were kept in standard polycarbonate cages (two animals per cage) containing autoclaved wood-chip bedding, which was changed three times per week to ensure hygiene. Animals had free access to tap water and were provided with a standard laboratory rat diet *ad libitum*.

To eliminate hormonal variability that might influence inflammatory responses or tissue healing, the oestrus cycles of all animals were evaluated via vaginal cytology for at least seven consecutive days before the interventions. Cycle synchronization was achieved using a hormonal regulation protocol based on previously established methods^1,5^. Only animals demonstrating successful oestrus synchronization were included in the surgical phase of the study.

All procedures were conducted in accordance with the Guide for the Care and Use of Laboratory Animals and institutional guidelines. Animals were acclimatized to the laboratory environment for at least one week before the initiation of experimental procedures to reduce stress-related physiological alterations.

### Surgical procedure

Following acclimatization and oestrus synchronization, 40 rats were randomly allocated into four experimental groups (n = 10 per group) using a computer-generated randomization list:

- Group A: polypropylene mesh implantation — acute period;
- Group AS: steroid-impregnated polypropylene mesh — acute period;
- Group C: polypropylene mesh implantation — chronic period;
- Group CS: steroid-impregnated polypropylene mesh — chronic period.

All animals were fasted for eight hours prior to surgery but allowed free access to water. Anaesthesia was induced with ketamine 50 mg/kg intraperitoneally. Anaesthetic depth was confirmed by absence of the pedal withdrawal, tail-pinch reflexes and loss of corneal reflex prior to the procedure. During the procedure, rats were placed on a heating pad to maintain normothermia and prevent hypothermia-related complications.

The abdominal area was shaved and disinfected with 10% povidone–iodine solution. A 3-cm midline vertical laparotomy was performed under sterile surgical conditions to access the peritoneal cavity. In groups A and C, a 1 × 1-cm polypropylene mesh (Gynemesh, Ethicon, Somerville, NJ, United States of America) was positioned on the right rectus abdominis muscle. Fixation was achieved using 3-0 polypropylene sutures (Prolene, Ethicon, Somerville, NJ, United States of America) with four interrupted stitches at the corners to ensure stable attachment and prevent mesh migration.

In groups AS and CS, a 1 × 1-cm steroid-impregnated polypropylene mesh was similarly placed and secured. These meshes were prepared immediately before implantation by immersing the polypropylene material in 50-mg methylprednisolone acetate solution for 1 hour under sterile conditions. Based on the mesh size and full absorption assumptions, the local steroid load was estimated to be 2.5 mg/cm^3^ per 1 × 1-cm mesh, consistent with previous methodologies^5,6^.

Following mesh placement, the abdominal wall was closed using 3-0 polyglactin 910 (Vicryl, Ethicon) in a continuous interlocking pattern. Skin closure was performed without tension to minimize postoperative dehiscence. Perioperative analgesia was provided by adding paracetamol at 6 mg/mL to the drinking water for 72 hours postoperatively.

Postoperative monitoring included assessment of activity, wound integrity, food and water intake, and signs of distress. No prophylactic antibiotics were administered to avoid confounding inflammatory outcomes.

Rats in acute groups (A and AS) underwent re-laparotomy at 72 hours post-implantation. After anaesthesia with ketamine 50 mg/kg intraperitoneal, the previously implanted meshes were carefully dissected, excised *en bloc* with surrounding tissue, and immediately fixed in 10% neutral buffered formalin for 24 hours.

Rats in chronic groups (C and CS) underwent the same retrieval procedure at nine weeks post-implantation, allowing chronic-phase tissue reactions to be evaluated. After mesh retrieval, all rats were humanely sacrificed using an anaesthetic overdose of ketamine 300 mg/kg intraperitoneal and xylazine 30 mg/kg intraperitoneal in accordance with ethical guidelines^1,5^.

### Histopathological evaluation

Following exploration, tissue samples containing the mesh and surrounding reaction zone were immediately fixed in 10% neutral buffered formalin for 24 hours. After fixation, samples were processed through graded ethanol solutions for dehydration, cleared in xylene, and embedded in paraffin blocks using a standardized automatic tissue processor. From each block, 5-µm-thick serial sections were obtained using a rotary microtome and mounted on positively charged glass slides to improve tissue adhesion during staining.

For general morphological assessment, sections were stained with hematoxylin and eosin (H&E). To evaluate the degree of fibrosis and collagen deposition, additional serial sections were stained using Masson’s trichrome according to standard protocols. All stained slides were examined under a light microscope (Nikon Eclipse E600W, Japan), and digital photomicrographs were obtained when necessary. A single experienced pathologist, blinded to the treatment groups and study design, performed all evaluations to avoid observer bias.

A semi-quantitative grading scale was used to assess inflammatory and tissue-reaction parameters in H&E-stained sections, including leukocyte infiltration, neovascularization, foreign body giant cell reaction, fibrosis, muscle invasion, necrosis of epithelial or surrounding tissue structures.

Each parameter was scored on a 0–3 scale:

- 0 = no change;
- 1 = mild;
- 2 = moderate;
- 3 = severe.

Higher scores indicated greater tissue disruption or inflammatory response.

For fibrosis intensity assessment, Masson’s trichrome–stained sections were analyzed following the methodology described by Erdemir et al.^6^. Collagen fiber density and organization around the mesh were evaluated at ×400 magnification and categorized into four grades:

- 1+ = minimal collagen deposition,
- 2+ = mild,
- 3+ = moderate,
- 4+ = intense and dense collagen deposition, representing the highest level of fibrosis^3,7^.

To ensure reproducibility, at least five randomly selected high-power fields per section were evaluated, and mean scores were calculated for each sample. All assessments adhered to standard histopathological criteria used in mesh-associated tissue response studies.

### Immunohistochemistry

Immunohistochemical analyses were performed on serial 5-µm paraffin-embedded tissue sections obtained from each sample. Sections were mounted on positively charged slides to improve tissue adherence during the staining process. Prior to staining, slides were incubated at 60°C for 1 hour to promote proper bonding of tissue to the glass.

Tissue sections were deparaffinized in xylene and rehydrated through graded alcohols to distilled water. Antigen retrieval was performed using heat-induced epitope retrieval in citrate buffer (pH 6.0) in a microwave oven for 20 minutes, followed by cooling to room temperature. Endogenous peroxidase activity was blocked using 3% hydrogen peroxide for 10 minutes to prevent nonspecific background staining.

Primary antibodies were applied as follows:

- CD3 (pan–T lymphocyte marker; 1:150 dilution; Santa Cruz Biotechnology, Heidelberg, Germany);
- CD15 (granulocyte marker; Santa Cruz Biotechnology, Heidelberg, Germany);
- CD20 (B-lymphocyte marker; Santa Cruz Biotechnology, Heidelberg, Germany);
- CD68 (pan-macrophage marker; 1:1,600 dilution, clone KP-1; DAKO, Glostrup, Denmark).

All biopsies were stained simultaneously in batched runs to minimize inter-assay and inter-staining variability. Primary antibody incubation lasted 32 minutes at room temperature in a humidity-controlled chamber. After rinsing in phosphate-buffered saline (PBS), sections were incubated with a horseradish peroxidase (HRP)-linked secondary antibody system using a commercially available detection kit. The chromogenic reaction was developed with 3,3’-diaminobenzidine (DAB), resulting in a brown cytoplasmic or membranous signal depending on the antigen. Slides were counterstained with Mayer’s hematoxylin, dehydrated, cleared, and cover slipped with a permanent mounting medium.

All specimens were examined under a light microscope (Nikon Eclipse E600W, Japan). For each slide, five randomly selected high-power fields at ×200 magnification were evaluated by a pathologist blinded to group assignments^1,3,5^. Immunostaining intensity and distribution for each marker were assessed semi-quantitatively to characterize the inflammatory cell composition at the mesh–tissue interface.

### Statistical analysis

Prior to hypothesis testing, the distribution characteristics of continuous variables were assessed using Shapiro–Wilk’s test, given the relatively small sample size in each group (n = 10). Variables that did not meet assumptions of normality were analyzed using non-parametric methods. Quantitative data were expressed as mean ± standard deviation, while qualitative variables were presented as frequencies and percentages. For overall intergroup comparisons among the four study groups (A, AS, C, and CS), the Kruskal–Wallis’ test was applied due to the ordinal nature of histopathological and immunohistochemical scoring. When the Kruskal–Wallis’ test indicated a significant overall difference, pairwise post-hoc analyses were performed using the Mann–Whitney’s U test with Bonferroni-adjusted p-values to control for type I error. Intragroup comparisons, when applicable, were evaluated using Tukey’s honestly significant difference (HSD) test following one-way analysis of variance (ANOVA) for normally distributed variables; for non-normal data, equivalent non-parametric post-hoc procedures were applied. Effect sizes were calculated when relevant to assess the magnitude of group differences. All statistical analyses were performed using the Statistical Package for the Social Sciences (SPSS), version 20.0 (SPSS Inc., Chicago, IL, United States of America). All statistical tests were two-tailed, and the level of statistical significance was set at *p* < 0.05.

## Results

The mean initial body weights of the rats after randomization were 271.4, 269.8, 270.7, and 272.1 g in groups A, AS, C, and CS, respectively. One rat in group CS died, and no postoperative complications or adverse events were observed in the remaining animals throughout the study.

Histopathological analysis revealed that polymorphonuclear leukocyte (PMNL) infiltration, neovascularization, foreign-body giant cell reaction, muscle invasion, and tissue necrosis did not significantly differ among the groups (*p* = 0.608, *p* = 0.264, *p* = 0.608, *p* = 0.614, *p* = 0.583, respectively; Table 1). In contrast, fibrosis was significantly higher in both chronic period groups (group C and group CS) compared to group A (*p* = 0.004, *p* = 0.004, respectively). Furthermore, group CS exhibited higher fibrosis levels than group AS (*p* = 0.001), indicating an enhanced fibrotic response associated with both chronic implantation and steroid impregnation.

**Table 1:** Comparison of the histopathological injury scores among groups.

	Group A	Group AS	Group C	Group CS	*p* -value
Inflammatory cell infiltration	2.4 ± 0.51	2.5 ± 0.52	2.2 ± 0.91	2.11 ± 0.51	0.608
Neovascularization	2.4 ± 0.51	2.5 ± 0.52	2 ± 0.81	2 ± 0.7	0.264
Foreign-body reaction	2.1 ± 0.73	2.2 ± 0.63	2.2 ± 0.63	1.88 ± 0.33	0.608
Fibrosis	0.9 ± 0.56	0.8 ± 0.42	1.8 ± 0.42	2 ± 0.7	< 0.01*
Muscle invasion	0.3 ± 0.48	0.3 ± 0.48	0.5 ± 0.52	0.22 ± 0.44	0.614
Necrosis	0.1 ± 0.31	0.1 ± 0.31	−	−	0.583

* *p* < 0.05; Kruskal-Wallis’ test; Tukey’s honest significant difference test. Intragroup comparisons for polymorphonuclear leukocyte infiltration; group A-group AS: (U = 45, *p* = 0.739); group A-group C: (U = 46,*p* = 0.796); group A-group CS: (U = 34, *p* = 0.400); group AS-group C: (U = 42.5, *p* = 0.579); group AS-group CS: (U = 30, *p* = 0.243); group C-group CS: (U = 40.5, *p* = 0.720). Intragroup comparisons for neovascularization; group A-group AS: (U = 45, *p* = 0.739); group A-group C: (U = 36, *p* = 0.315); group A-group CS: (U = 31, *p* = 0.278); group AS-group C: (U = 32.5, *p* = 0.190); group AS group CS: (U = 27.5, *p* = 0.156); group C-group CS: (U = 45, *p* > 0.05). Intragroup comparisons for foreign-body reaction; group A-group AS: (U = 46.5, *p* = 0.796); group A-group C: (U = 46.5, *p* = 0.796); group A-group CS: (U = 37, *p* = 0.549); group AS-group C: (U = 50, *p* > 0.05); group AS-group CS: (U = 32.5, *p* = 0.315); group C-group CS: (U = 32.5, *p* = 0.315). Intragroup comparisons for fibrosis; group A-group AS: (U = 45, *p* = 0.796); group A-group C: (U = 13, *p* = 0.004); group A-group CS: (U = 11.5, *p* = 0.004); group AS-group C: (U = 8, *p* = 0.001); group AS-group CS: (U = 8, *p* = 0.001); group C-group CS: (U = 38, *p* = 0.604). Intragroup comparisons for muscle invasion; group A-group AS: (U = 45, *p* > 0.05); group A-group C: (U = 40, *p* = 0.481); group A-group CS: (U = 41.5, *p* = 0.780); group AS-group C: (U = 40, *p* = 0.481); group AS-group CS: (U = 41.5, *p* = 0.780); group C-group CS: (U = 32.5, *p* = 0.315). Intragroup comparisons for necrosis; group A-group AS: (U = 45, *p* > 0.05); group A-group C: (U = 45, *p* = 0.739); group A-group CS: (U = 40.5, *p* = 0.720); group AS-group C: (U = 45, *p* = 0.739); group AS-group CS: (U = 40.5, *p* = 0.720); group C-group CS: (U = 45, *p* > 0.05).

Source: Elaborated by the authors.

Comparison of the percentage change in histopathological parameters between steroid-impregnated polypropylene mesh and polypropylene mesh revealed no statistically significant differences (Table 2). The degree of PMNL infiltration was comparable between the groups (-8.33 *versus* -7.41%, respectively; *p* = 0.905). Similarly, the reduction in neovascularization showed no meaningful distinction (-16.67 *versus* -12.96%, respectively; *p* = 0.905). Foreign-body reaction demonstrated a greater percentage increase in polypropylene mesh group compared with steroid-impregnated polypropylene mesh group (21.67 *versus* -5.56%, respectively; *p* = 0.356). Fibrosis showed a higher percentage increase in steroid-impregnated polypropylene mesh group compared with polypropylene mesh group (100 *versus* 75%, respectively; *p* = 0.613). Muscle invasion and necrosis were not observed in either group.

**Table 2:** The difference in percentage between mesh without steroid and mesh with steroid application.

	No steroids	With steroids	*p* -value*
Inflammatory cell infiltration	-8.33 ± 37.88	-7.41 ± 39.18	0.905
Neovascularization	-16.67 ± 32.39	-12.96 ± 41.48	0.905
Foreign-body reaction	21.67 ± 73.72	-5.56 ± 44.09	0.356
Fibrosis	75 ± 46.29	100 ± 81.65	0.613
Muscle invasion	−	−	−
Necrosis	−	−	−

* *p* < 0.05; Mann-Whitney’s U test. Source: Elaborated by the authors.

**Figure 1:**
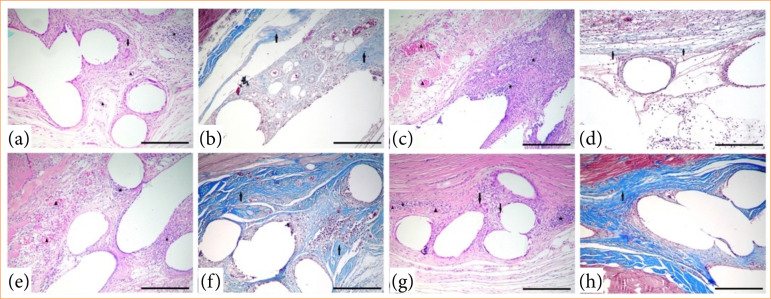
The histopathological evaluation of meshes. (a) Star indicates inflammatory cell infiltration, triangle shows vascular tissue (group A – polypropylene mesh in acute period); (b) up arrows show fibrosis (group A – polypropylene mesh in acute period); (c) Star indicates inflammatory cell infiltration, triangle shows vascular tissue (group AS – steroid impregnated polypropylene mesh in acute period); (d) up arrows show fibrosis (group AS – steroid impregnated polypropylene mesh in acute period); (e) star indicates inflammatory cell infiltration, triangle shows vascular tissue (group C – polypropylene mesh in chronic period); (f) up arrows show fibrosis (group C – polypropylene mesh in chronic period); (g) star indicates inflammatory cell infiltration, triangle shows vascular tissue, down arrows mention polymorphonuclear leucocytes (group CS – steroid impregnated polypropylene mesh in chronic period); (h) up arrow shows fibrosis (group CS – steroid impregnated polypropylene mesh in chronic period).

In the acute period, implantation of polypropylene mesh induced inflammatory changes in all groups. In group A, H&E staining demonstrated PMNL infiltration surrounding the mesh filaments, accompanied by identifiable vascular structures (Fig. 1a). Masson’s trichrome staining showed early collagen deposition and focal fibrosis (Fig. 1b). In group AS, steroid-impregnated mesh produced a similar but slightly less intense inflammatory infiltrate, with preserved vascular structures (Fig. 1c). Early fibrosis was observable on trichrome staining (Fig. 1d).

(a, c, e and g): hematoxylin and eosin staining, magnification: ×100, scale bar: 200 micrometers; (b, d, f and h) Masson’s trichrome staining, magnification: ×100, scale bar: 200 micrometers. Source: Elaborated by the authors.

In the chronic period, group C exhibited persistent PMNL infiltration and mature vascular tissue on H&E staining (Fig. 1e), with more organized fibrotic tissue visible on Masson’s trichrome (Fig. 1f). Group CS showed reduced inflammatory infiltrate, with only scattered PMNL and well-defined vascular structures (Fig. 1g). Corresponding trichrome staining demonstrated mature but comparatively less dense collagen deposition (Fig. 1h).

Across both periods, steroid-impregnated meshes (groups AS and CS) displayed qualitatively reduced inflammatory infiltration and less prominent fibrosis compared with non-steroid groups (Figs. 1a and 1c). Neovascularization was present in all groups without marked qualitative differences.

**Figure 2:**
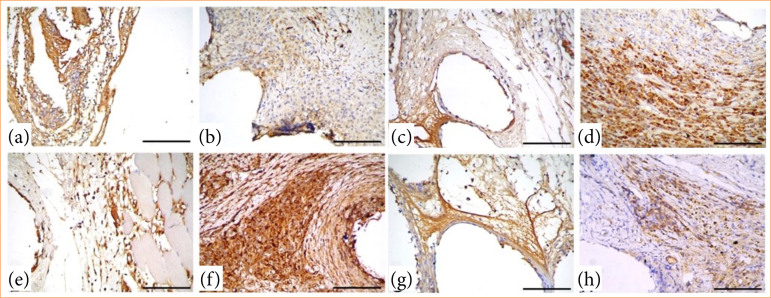
Immunohistopathological evaluation of the meshes. (a) Immunohistochemical staining for CD15 of group AS (steroid impregnated polypropylene mesh in acute period); (b) immunohistochemical staining for CD15 of group CS (steroid impregnated polypropylene mesh in chronic period); (c) immunohistochemical staining for CD68 of group AS (steroid impregnated polypropylene mesh in acute period); (d) immunohistochemical staining for CD68 of group CS (steroid impregnated polypropylene mesh in chronic period); (e) immunohistochemical staining for CD3 of group AS (steroid impregnated polypropylene mesh in acute period); (f) immunohistochemical staining for CD3 of group CS (steroid impregnated polypropylene mesh in chronic period); (g) immunohistochemical staining for CD20 of group AS (steroid impregnated polypropylene mesh in acute period); (h) immunohistochemical staining for CD20 of group CS (steroid impregnated polypropylene mesh in chronic period).

Immunohistochemical analysis revealed distinct inflammatory and immune-cell profiles around the polypropylene meshes in both acute and chronic periods. In the acute period, CD15 staining demonstrated pronounced neutrophilic infiltration in group AS (Fig. 2a), whereas a comparatively less intense CD15 response was observed in group CS (Fig. 2b). CD68 expression, indicative of macrophage activity, was prominent in group AS during the acute period (Fig. 2c), showing dense macrophage accumulation around the mesh fibers. In the chronic phase, CD68-positive macrophages remained abundant in group CS (Fig. 2d), suggesting ongoing foreign-body–type inflammation.

Magnification: ×200, scale bar: 100 micrometers. Source: Elaborated by the authors.

T-lymphocyte infiltration, assessed by CD3 staining, showed moderate perivascular and perimesh distribution in group AS during the acute period (Fig. 2e), while group CS exhibited a more extensive and diffuse CD3-positive T-cell presence in the chronic phase (Fig. 2f). B-lymphocyte activity, evaluated by CD20 staining, was relatively limited in group AS during the acute period (Fig. 2g), whereas chronic-phase tissue from group CS showed scattered but detectable CD20-positive cells (Fig. 2h), consistent with a low-grade adaptive immune response.

## Discussion

The present study evaluated the effects of steroid-impregnated polypropylene meshes on tissue responses in both acute and chronic periods. Our results demonstrated that local corticosteroid application did not significantly alter inflammatory cell infiltration, neovascularization, foreign body reaction, muscle invasion, or necrosis compared to standard polypropylene meshes. The only parameter showing a clear temporal change was fibrosis, which—consistent with normal wound healing physiology—progressively increased from acute to chronic period^1,2,6^. Immunohistochemical findings supported the histopathological observations: granulocyte and B-cell infiltrates were more localized in the acute phase and became more dispersed in chronic samples, while T-lymphocyte and macrophage densities increased over time^1,5^. These changes reflect the expected transition from acute inflammation toward chronic, macrophage-driven remodeling.

Experimental evidence supports the concept that locally delivered corticosteroids or surface modifications can modulate the host response to implanted meshes. Brandt et al.^7^ demonstrated that hydrocortisone- or spironolactone-coated polyvinylidene fluoride meshes reduced inflammatory activity and promoted more favorable long-term tissue remodeling in a mouse model, likely through early glucocorticoid and mineralocorticoid receptor activation. Similarly, Wolf et al.^8^ showed that bioactive surface coatings on polypropylene meshes can attenuate early adhesion formation and inflammatory cell recruitment, although these benefits tend to diminish with prolonged implantation. More recently, Li et al.^9^ reported that dexamethasone mitigates mesh-induced inflammatory responses by downregulating miR-155 and its downstream JAK1/STAT3 signaling pathway, providing mechanistic support for the anti-inflammatory effects of corticosteroid-based strategies. Collectively, these studies suggest that both receptor-mediated and molecular pathway–specific mechanisms may contribute to the reduced inflammatory profile observed with steroid-treated or coated mesh materials.

In the present study, the modest attenuation of inflammatory parameters observed in the steroid-impregnated groups during the chronic phase did not translate into a meaningful reduction in fibrosis severity. In fact, fibrosis scores in these groups more than doubled compared with the acute phase, indicating a paradoxical progression of tissue remodeling despite early steroid exposure. Several mechanistic explanations may account for this outcome:

- The administered corticosteroid dose (2.5 mg per 1×1-cm mesh) may have been insufficient to achieve sustained pharmacological activity;
- Rapid dissolution and clearance of the impregnated steroid likely limited its effective duration of action;
- Local corticosteroid delivery alone may be inadequate to maintain long-term modulation of wound-healing pathways;
- The chronic phase is dominated by fibroblast-mediated extracellular matrix deposition, a process potentially less amenable to short-term glucocorticoid suppression.

Collectively, these factors may explain why early anti-inflammatory effects did not confer durable antifibrotic benefit.

Fibroblast migration begins as early as two to five days after injury, and by the end of the first week fibroblasts become the dominant cell population in the wound environment. Corticosteroids are known to inhibit fibroblast proliferation and collagen matrix formation, but this effect is dose dependent. Recent animal studies provide additional context for understanding the variable effects of corticosteroids on tissue healing and fibrosis. In a rat mucosal-injury model, Weinberg et al.^10^ administered systemic dexamethasone either early (days 1–4) or later (days 5–9) during healing and found no significant improvement in wound closure; by three weeks, steroid-treated defects remained larger and histological inflammation scores did not differ from controls. Similarly, Kafa et al.^11^ reported that local dexamethasone altered inflammatory and extracellular matrix–related gene expression in a rat Achilles tendon–healing model but ultimately impaired tendon-construct formation and collagen organization, particularly under mechanical loading. In contrast, Doci et al.^12^ demonstrated that triamcinolone incorporated into surgical glue reduced neovascularization and early fibrosis in rat cutaneous wounds and produced a looser, less compact collagen matrix at days 7 and 14.

Collectively, these studies indicate that while corticosteroids can modulate early inflammatory or remodeling pathways, their long-term effects on tissue regeneration are inconsistent and highly dependent on tissue type, dosage, route of administration, and biomechanical environment. The chronic-phase fibrosis observed in this study suggests that the locally administered corticosteroid dose may have been below the level required to modify fibroblast-driven remodeling or extracellular matrix synthesis. These findings support the interpretation that the limited chronic antifibrotic response observed in the present study may reflect the inherent limitations of corticosteroid-based modulation in mesh-associated wound healing.

Despite extensive research, the biological response to implanted alloplastic materials remains complex^10-13^. Early inflammatory cell recruitment at the implant site transitions into macrophage-driven remodeling, ultimately dominated by fibroblast proliferation and collagen deposition. These stages collectively influence adhesion formation and mesh biocompatibility^11,13-16^. Animal studies therefore remain crucial in elucidating the interactions between synthetic implants and host tissues.

This study presents several limitations that should be acknowledged. First, the number of animals in each experimental group was relatively small, which may have reduced the statistical power to detect modest differences in histopathological or immunohistochemical responses. Although the loss of one rat in the chronic steroid-impregnated mesh group did not preclude analysis, it may have affected intergroup balance. Second, tissue responses were assessed at only two discrete time points—acute and chronic—without intermediate evaluations. More frequent sampling could have provided a finer temporal resolution of inflammatory dynamics, neovascularization, and fibrosis development. Third, the study relied on conventional qualitative and semi-quantitative histopathological scoring systems. Despite their widespread use, these methods are susceptible to inter-observer variability. Incorporating automated digital pathology, morphometric analysis, or quantitative molecular assays would improve accuracy and reproducibility. The immunohistochemical panel (CD15, CD68, CD3, CD20) was also limited. Inclusion of additional markers related to extracellular matrix remodeling, macrophage polarization, or cytokine signaling could offer a more comprehensive characterization of tissue–mesh interactions. Finally, although the rat abdominal wall model is well established, it does not fully replicate the biomechanical and immunological conditions encountered in human hernia repair. Differences in mesh integration, tissue loading, and immune responsiveness restrict the direct translation of these findings to clinical practice.

## Conclusion

Our findings indicated that local corticosteroid impregnation of polypropylene meshes at a dose of 2.5 mg methylprednisolone does not sufficiently reduce chronic fibrotic tissue reactions, although a mild attenuation of early inflammatory responses was observed. These results suggest that higher local doses, sustained-release steroid coatings, or combined systemic and local corticosteroid strategies may be necessary to achieve meaningful modulation of the foreign-body response. Future studies should also explore long-term outcomes, dose–response relationships, and the potential of multi-layered or bioactive mesh coatings to enhance biocompatibility and reduce postoperative complications.

## Data Availability

The datasets used or analyzed during the current study are available from the corresponding author on reasonable request.
